# The timing, duration and magnitude of the 8.2 ka event in global speleothem records

**DOI:** 10.1038/s41598-022-14684-y

**Published:** 2022-06-22

**Authors:** Sarah E. Parker, Sandy P. Harrison

**Affiliations:** School of Archaeology, Geography and Environmental Science, Reading University, Whiteknights, Reading, RG6 6AH UK

**Keywords:** Climate change, Palaeoclimate

## Abstract

Abrupt events are a feature of many palaeoclimate records during the Holocene. The best example is the 8.2 ka event, which was triggered by a release of meltwater into the Labrador Sea and resulted in a weakening of poleward heat transport in the North Atlantic. We use an objective method to identify rapid climate events in globally distributed speleothem oxygen isotope records during the Holocene. We show that the 8.2 ka event can be identified in >70% of the speleothem records and is the most coherent signal of abrupt climate change during the last 12,000 years. The isotopic changes during the event are regionally homogenous: positive oxygen isotope anomalies are observed across Asia and negative anomalies are seen across Europe, the Mediterranean, South America and southern Africa. The magnitude of the isotopic excursions in Europe and Asia are statistically indistinguishable. There is no significant difference in the duration and timing of the 8.2 ka event between regions, or between the speleothem records and Greenland ice core records. Our study supports a rapid and global climate response to the 8.2 ka freshwater pulse into the North Atlantic, likely transmitted globally via atmospheric teleconnections.

## Introduction

The Holocene epoch (11,700 years BP to present) has been punctuated by several large-scale and rapid changes in the climate system^1–3^, termed abrupt events. Numerous abrupt events have been identified, although many have not been studied extensively or have only been identified in a limited number of regions, and the causes of the events are not always clear. Two events that have been studied and examined more extensively are the 4.2 and 8.2 ka (ka; thousand years ago) events. The 4.2 ka event is a 300-year megadrought identified predominantly in Eurasian and Middle Eastern palaeoclimate records^4–6^, although the exact mechanism is still debated^7,8^.

The largest and most-significant abrupt event of the last 12,000 years in Greenland ice core records is the 8.2 ka event^9^. During this event, an influx of freshwater into the Labrador Sea from the retreating Laurentide ice sheet slowed down the Atlantic Meridional Overturning Circulation (AMOC), reducing northwards meridional heat transport^10,11^. This triggered a large drop in temperature across the North Atlantic region; Greenland ice cores show > 2 °C cooling over an interval of 165 years^12^. The 8.2 ka event has been identified in a large number of palaeoclimate records. A global compilation of reconstructions using marine, lake, ice and peat cores and speleothem records by Morrill et al.^13^ showed widespread cooling over Europe of ~ 1 to 1.5 °C. Drier conditions were shown in the northern hemisphere tropics, with wetter conditions in the southern hemisphere tropics. While this compilation has been used to evaluate climate model simulations of the 8.2 ka event^14–16^, only 13% of the records provide quantitative estimates of temperature and precipitation and most of the information consists of qualitative indications of the direction of the change in climate. Furthermore, most of the records included in this compilation were of insufficient temporal resolution to estimate the duration of the event, and the exact timing of the event was also not examined. Questions therefore remain about the global signature and nature of this event.

Speleothem oxygen isotope (δ^18^O) records are ideal for reconstructing global-scale patterns of abrupt climate events, such as the 8.2 ka event, because they often have sub-annual to decadal temporal resolution and well-constrained chronologies. Speleothem δ^18^O can be influenced by multiple climate factors, including regional precipitation, atmospheric circulation and temperature^17–19^, which can make their climate interpretation challenging. Nevertheless, the 8.2 ka event has been identified in numerous individual speleothem records around the world^20–23^ and there are now sufficient numbers of published speleothem records, especially compared to the status at the time of the Morrill et al.^13^ 8.2 ka synthesis, to facilitate a global-scale analysis^24^.

Here, we used the Speleothem Isotope Synthesis and AnaLysis (SISAL) database^25,26^ to identify potential abrupt climate events in the Holocene, by using breakpoint analysis to detect shifts in δ^18^O values objectively and determining whether these excursions were above the inherent variability of climate and spatially coherent. We then focus on the nature of the 8.2 ka event, and specifically the timing, duration and magnitude of the anomalies registered in each speleothem record at this time. We compare our speleothem synthesis to other lines of evidence, including Greenland ice core data, speleothem trace element records and the global synthesis by Morrill et al.^13^. Our new global synthesis allows us to address the following questions: (1) Is the 8.2 ka event a significant and prominent feature of the Holocene epoch? (2) How rapidly was the event transmitted to regions distant from the North Atlantic? (3) What is the speleothem δ^18^O spatial fingerprint of this event, and what does it tell us about the climate response?

## Results

**Figure 1:**
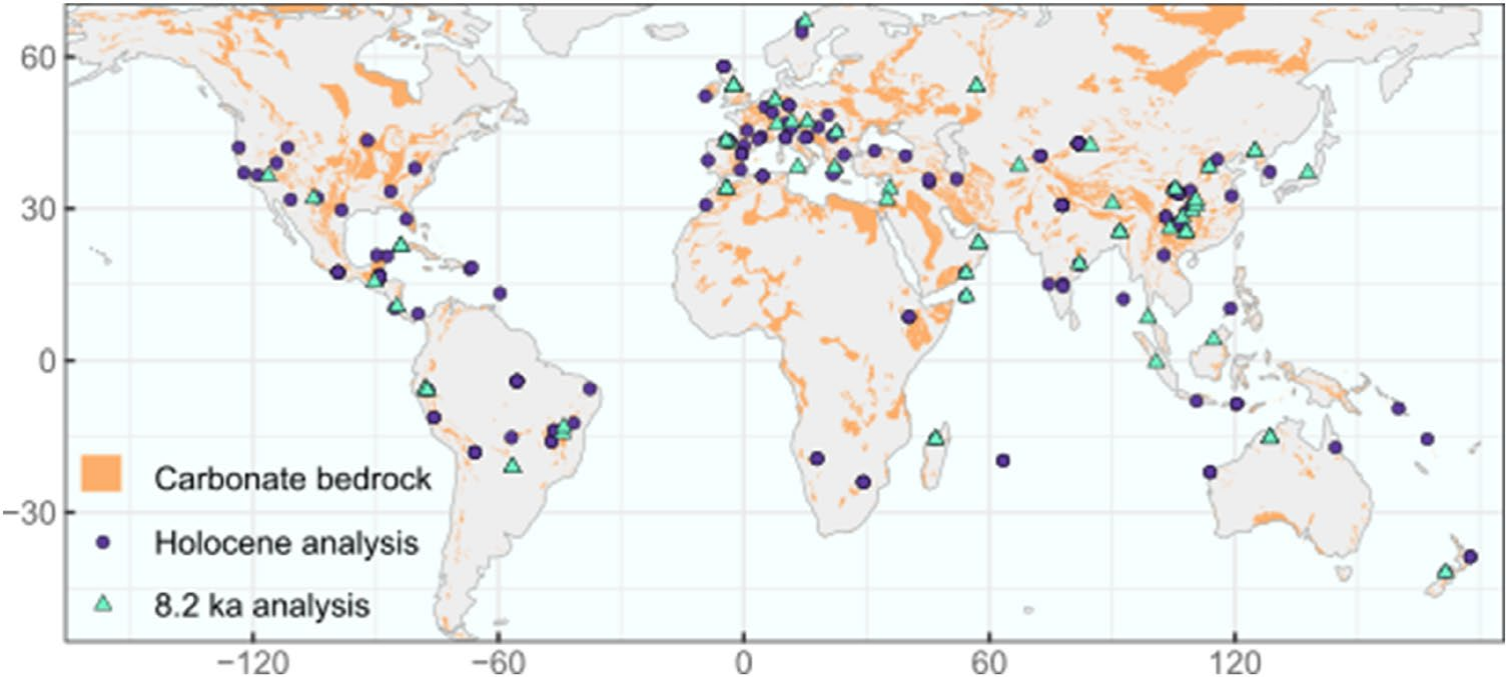
Spatial distribution of speleothem δ^18^O records used in this study. Purple dots show sites that were only used to detect globally significant abrupt events through the Holocene, and green triangles show those used both in the Holocene analysis and to examine the 8.2 ka event. Carbonate bedrock distribution is from the WOrld Karst Aquifer Mapping (WOKAM) project^67^.

**Figure 2:**
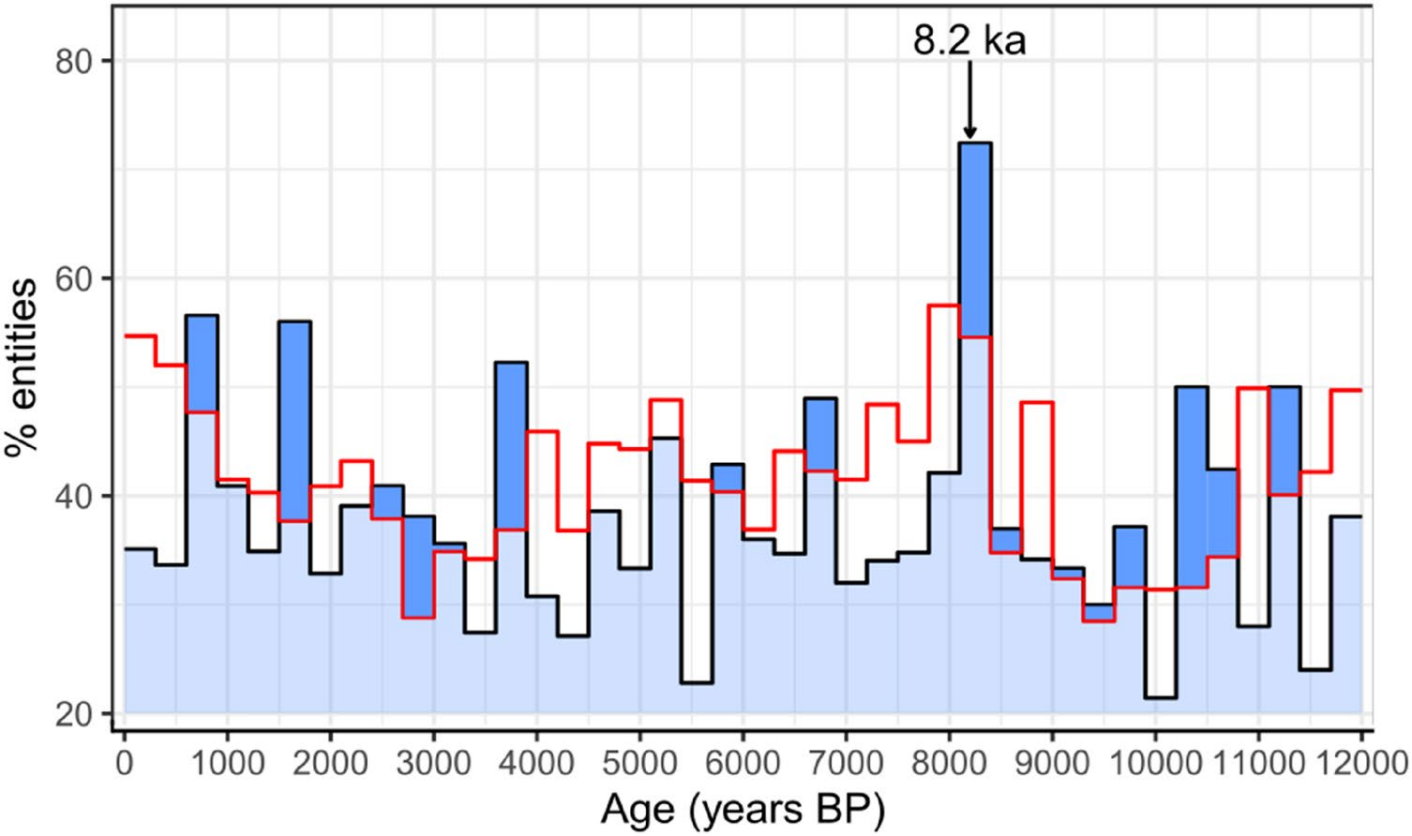
Percentage of high-resolution speleothem records that show at least two abrupt shifts in oxygen isotope values, for a given 300-year bin, across the Holocene epoch. The 8.2 ka event is annotated. The red line represents the percentage of randomly generated records (with red noise) that show >  = 2 breakpoints in a 300-year bin. The bins that are higher than the randomly generated noise (and therefore significant) are shown in dark blue.

We examined the presence and timing of significant abrupt climate events through the Holocene using 275 globally distributed speleothem records (Fig. 1). There are several intervals where the proportion of records showing an abrupt isotope excursion exceeds the randomly generated noise (Fig. 2), including at 0.6–0.9, 1.5–1.8, 3.6–3.9, 6.6–6.9, 8.1–8.4, 10.2–10.5 and 11.1–11.4 ka. The early Holocene peaks coincide with Bond event 7 (10.3 ka) and 8 (11.1 ka, or the pre-Boreal oscillation)^27,28^. However, the isotope excursions associated with each of these two peaks show little coherency in their spatial pattern, timing and duration (Fig. S1). The excursions identified between 8 ka and present do not coincide with any previously identified abrupt climate events. Although the interval around 4.2 ka is often identified as a period of rapid climate change^4–6^, it is not detected by the speleothem records. The most prominent period of abrupt climate change in the Holocene is at ~ 8.2 ka, where 72% of the records show an abrupt isotope excursion.

**Figure 3:**
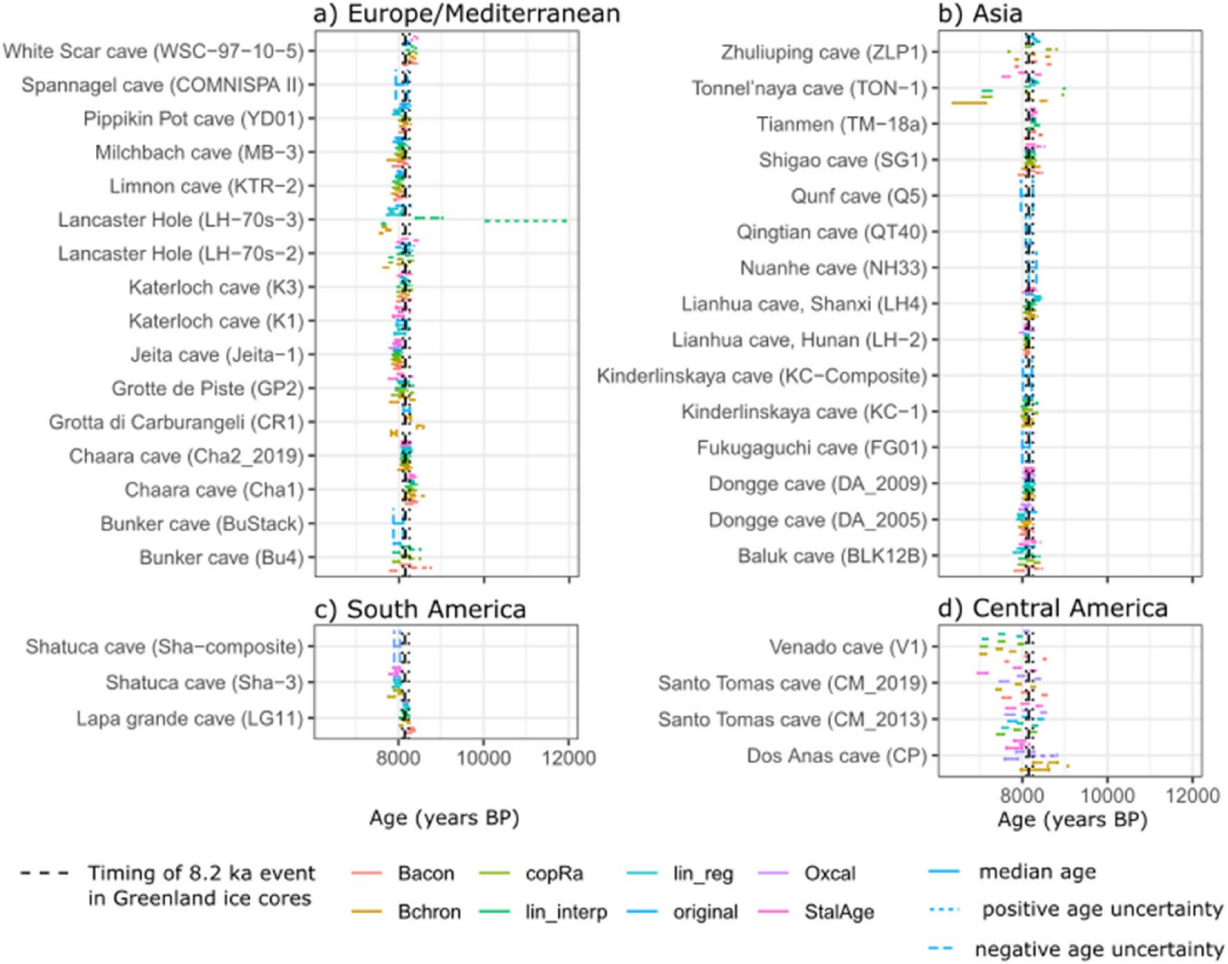
Start and end of the 8.2 ka event for each record and each age-depth modelling approach, constrained by breakpoint analysis. The original chronologies are shown together with the SISALv2 chronologies. Upper and lower age uncertainties associated with each age-depth model are given (dashed lines). The 8.2 ka event timing in the Greenland ice core record is shown^12^.

We examined the isotope excursion at 8.2 ka using 73 speleothem δ^18^O records (Fig. 1) of sufficiently high temporal resolution and length (see “Methods” section). Most records show a remarkably consistent timing (Fig. 3) of the event, allowing for age uncertainties. The global speleothem records show the event starting at 8.22 ± 0.012 ka and ending at 8.06 ± 0.014 ka (Table 1). Furthermore, the timing of the global δ^18^O excursion coincides with the 8.2 ka event excursion identified in Greenland ice core records, within age uncertainties^12^. The median duration registered globally in speleothem δ^18^O records (Table. 1) is ~ 159 years, which is the same (within error) as the duration of the event calculated by layer counting in Greenland ice cores (of 160.5 years^12^). Both the timing and the duration of the event are statistically indistinguishable between Europe and Asia, the two regions with sufficient records to perform a t-test, and between these two regions and Greenland. The median magnitude of the 8.2 ka δ^18^O excursion is also indistinguishable between Europe and Asia (Table 1).

**Table 1:** Median start, end and duration of the 8.2 ka event registered in speleothem records, globally and for the Europe/Mediterranean and Asia regions.

	Magnitude (permil)	Start (years BP)	End (years BP)	Duration (years)
Global	0.5 (0.04)	8223 (12)	8062 (14)	159 (11)
Europe	0.4 (0.05)	8192 (27)	7968 (33)	166 (22)
Asia	0.49 (0.07)	8257 (14)	8081 (16)	163 (15)
Greenland		8247 (47)	8086 (47)	160.5 (5.5)

Standard error associated with each value is given in brackets. For all variables, regional values are insignificant from one another, according to a t test (at *P* < 0.01). Timing and duration of the 8.2 ka event in Greenland ice core are also shown, with their uncertainty^9,12^.

**Figure 4:**
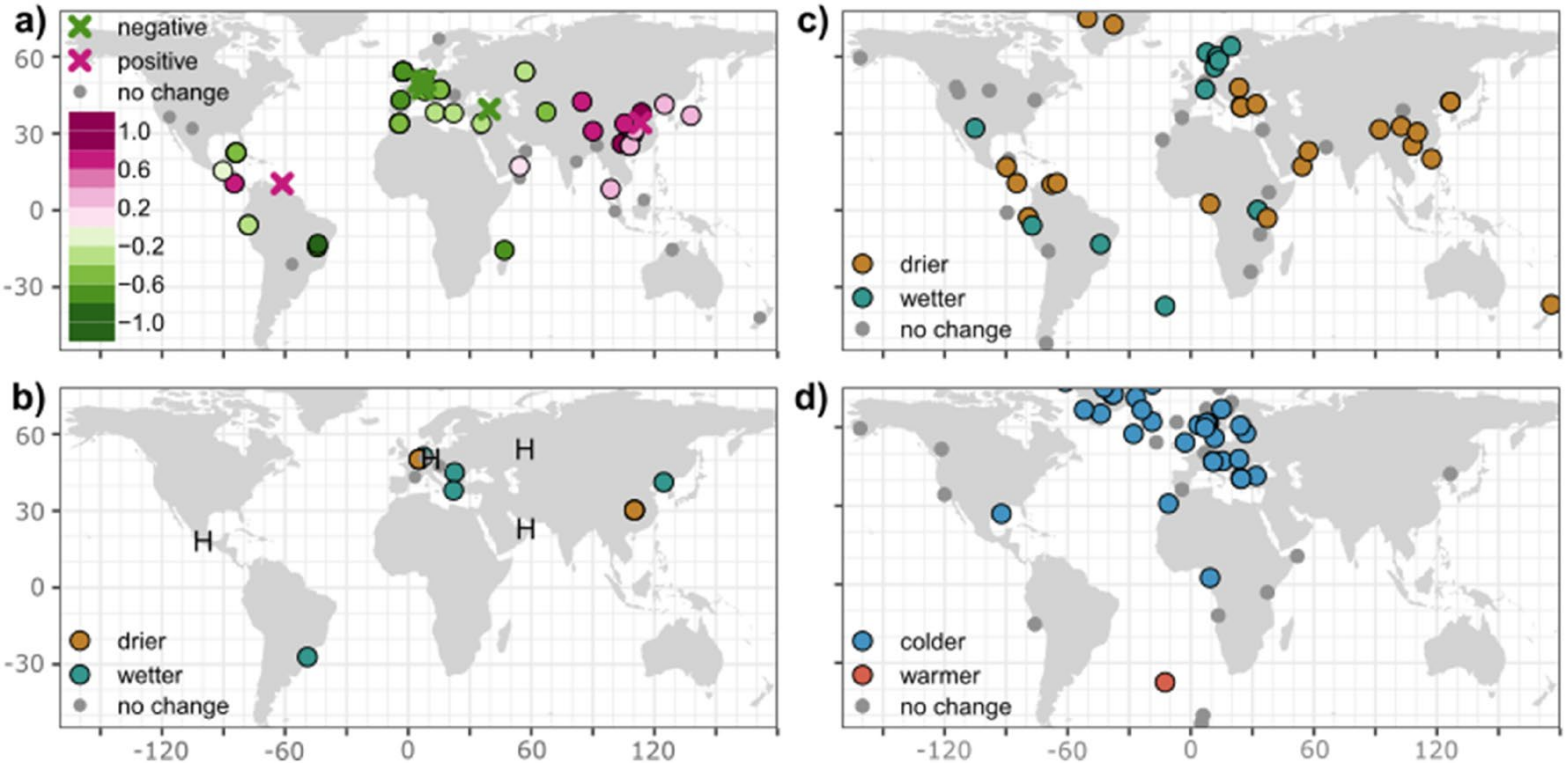
**(a)** Oxygen isotope anomalies for the 8.2 ka event, registered by speleothem records. The 8.2 ka anomalies determined by breakpoint analysis are shown by filled circles. They are calculated as the mean δ^18^O value between the start and end of the event and given relative to a base period mean δ^18^O, defined as 7.8 ka to the event end and the start to 8.4 ka. Where studies have identified a speleothem δ^18^O excursion, but the raw data are not available, anomalies are simply given as positive or negative (Table S2), represented by crosses. (**b)** Speleothem evidence of wetter/drier 8.2 ka event conditions, inferred from non-oxygen isotope evidence. H indicates records with a hiatus spanning the 8.2 ka event and therefore assumed to record drier conditions. Metadata for these records are given in Table S3. (**c, d)** Inferred precipitation and temperature anomalies during the 8.2 ka event from the Morrill et al.^13^ synthesis.

Speleothem δ^18^O anomalies of the 8.2 ka event show homogeneous signals over broad regions (Fig. 4a). Over Europe and the Mediterranean region, 15 out of 20 sites exhibit an 8.2 ka excursion. These excursions show negative 8.2 ka δ^18^O anomalies, i.e. δ^18^O values across the event are lower (more negative) than before or after. Over Asia, anomalies are registered in 13 out of 16 sites, with consistently higher (positive) δ^18^O anomalies, where δ^18^O across the event are less negative than before and after the event. Speleothem δ^18^O anomalies are negative across the South American continent and southern Africa, registered in 4 out of 6 sites. All the central American sites show an 8.2 ka isotope excursion, although more southerly sites show positive excursions and more northern sites show negative δ^18^O anomalies. However, these sites show larger age uncertainties (Fig. 3) than other regions and there is therefore some uncertainty associated with the identification of the 8.2 ka isotope excursion. There is no significant 8.2 ka isotope excursion recorded in any of the four sites located in the Oceania region or in the two sites from North America.

The patterns in the speleothem δ^18^O anomalies (Fig. 4a) can be compared with evidence from speleothem growth rate, trace element and calcium isotope data (Fig. 4b, Table S3) and the Morrill et al.^13^ 8.2 ka reconstructions. The homogenous negative speleothem isotope signals across Europe are mirrored by widespread cooling signals (Fig. 4d). However, the precipitation anomalies (Fig. 4c) inferred by Morrill et al.^13^ and indicated by other speleothem evidence (Fig. 4b) are heterogeneous over the region, and indeed differ from one another. This heterogeneity could reflect the fact that different climate archives record different aspects of the hydrological cycle. For example, wet anomalies in west Europe from the Morrill et al. synthesis were explained as reflecting increased runoff from spring snowmelt, whilst dry signals in the east were inferred from pollen-based reconstructions of annual precipitation. Site-specific influences may also be obscuring regional climate signals in some records. Nevertheless, precipitation patterns in Fig. 4b, c do not show the homogeneity characteristic of the δ^18^O anomalies. The widespread positive speleothem isotope anomalies over Asia are mirrored by dry anomalies over the region (Fig. 4c), inferred from speleothem trace elements, growth rate and calcium isotopes, and peat δ^13^C and a South China Sea salinity record^13^. There are very few high-resolution 8.2 ka records in South America beyond speleothem δ^18^O evidence, however a trace element record from Botuverá Cave (Brazil)^29^ suggests wetter conditions during the event (Fig. 3b;^29^), consistent with the negative δ^18^O anomalies in the region. Drier conditions are inferred over central America by Morrill et al.^13^, whereas the speleothem isotope signals show a more mixed signal.

## Discussion

The prominence of the 8.2 ka event in Greenland records is perhaps unsurprising, given that it was forced by a large change in freshwater flux to the North Atlantic^11,30,31^. Its prominence in the speleothem records shows that this change was sufficient to trigger a global reorganisation of the climate system. We have shown that the 8.2 ka event shows remarkable global coherency with respect to timing, duration, magnitude and spatial pattern. The coeval timing of the event in regions both close and far from the north Atlantic (Europe and the Mediterranean versus Asia) supports the indirect evidence of global synchronicity from Greenland ice core methane records^32^, which reflect hydrological changes over methane producing regions, mainly tropical wetlands. The 8.2 ka methane excursion is coeval (within 4 years) with changes in δ^14^N (which reflects changes in local temperature) in Greenland, indicating that the North Atlantic and global climate response to the 8.2 ka freshwater influx is indeed synchronous. The Greenland ice core δ^18^O record and a sub-annual resolution speleothem record from Heshang Cave, China were found to be statistically indistinguishable, supporting a rapid (annual) teleconnection between these regions^33^. Furthermore, a comparison of eight speleothem records from China, Oman and Brazil^34^ showed the event occurred at the same time in all the records, within the dating uncertainties. Oceanic teleconnections operate on decadal to centennial timescales^35^. Since lags on these timescales are not observed (even within uncertainties) between near (Europe) and far (Asia) regions, our study supports the idea that the transmission of the 8.2 ka event occurred through suitably rapid atmospheric processes.

One atmospheric mechanism for the transmission of the North Atlantic signal globally is a southward shift in the mean position of the intertropical convergence zone (ITCZ)^36^, in response to cooler sea-surface temperatures (SSTs) in the North Atlantic. Such a shift in the mean position of the ITCZ is supported by the spatial patterning of the speleothem isotope signals (Fig. 4a). The antiphase pattern of positive signals in the northern hemisphere tropics of Asia and negative signals in the southern hemisphere tropics of South America and southern Africa is consistent with the weakening of northern hemisphere monsoons and strengthening of southern hemisphere monsoons in response to a shift in the ITCZ. Negative (positive) speleothem isotope signals in the monsoon regions have been interpreted as reflecting a stronger (weaker) monsoon, via a combination of processes, including regional precipitation and atmospheric circulation changes driving moisture transport changes^37,38^. The antiphase pattern in the tropics is also evident in precipitation anomalies^13^ and other lines of speleothem evidence (Fig. 4b). Quantitative precipitation reconstructions from Chinese speleothem records also support a significant drying over Asia during the 8.2 ka event. A calcium isotope record from Heshang cave (China) show a ~ 1/3rd decrease in precipitation at the onset of the event^39^, whilst a rainfall reconstruction using the difference in speleothem δ^18^O between two Chinese cave sites along a moisture pathway indicate a maximum 24% decrease of rainfall at the onset (350 mm year^−1^)^40^. Drier conditions in the northern hemisphere tropics and wetter conditions in the southern hemisphere tropics are also simulated by numerous climate model simulations of the 8.2 ka event and attributed to a shift in the ITCZ^14,15^. Thus, both palaeoclimate observations and climate model simulations support the idea that the 8.2 ka event was transmitted to the low latitudes via a shift in the mean position of the ITCZ.

The lack of 8.2 ka signal in the Indonesia/Australia region is inconsistent with this antiphase pattern in the tropics. There is no evident signal in other hydrological palaeoclimate records in the region^41^ and climate models typically show a mixed and non-significant rainfall response there^16,42^. This likely reflects the complexity of climate variability in the region, with ocean feedbacks playing a significant role^43,44^. Over central America, the mixed speleothem isotope signal contrasts with the drying signal inferred from lake records^13^ and simulated by climate models^15,16,42^. It is possible that the negative δ^18^O anomalies observed in the northern sites reflect lower δ^18^O of seawater in the north of the Gulf of Mexico, observed in a marine δ^18^O record^45^. However, the age uncertainties of these speleothem records are larger than most records (Fig. 3) and there are other δ^18^O excursions at around this time. In the study documenting the Dos Anas and Santo Tomas records, an earlier positive δ^18^O anomaly is tentatively suggested as reflecting the 8.2 ka event^46^. More high-resolution speleothem records of the 8.2 ka event are needed in this region to understand the climate response in the central America region better.

The negative speleothem isotope anomalies over Europe are consistent with the widespread cooling observed in numerous palaeoclimate records^13^. Quantitative temperature estimates suggest a cooling of between 1 and 1.5 °C^13,47^. Based on the observed and modelled temperature/δ^18^O_precipitation_ gradients of 0.17‰ to 0.9‰ °C^−1^, and an equilibrium isotope fractionation between drip water and calcite of − 0.18 to − 0.23 ‰ °C^−1^ ^18^, the regional δ^18^O speleothem anomaly of 0.4 ‰ could be fully explained by the regional temperature decrease. The oxygen isotopic composition of moisture delivered to Europe was also likely lower during the 8.2 ka event, further contributing to negative isotope anomalies in the region. Lower δ^18^O of seawater (of ~ 0.4 ‰^48^) in the North Atlantic region and cooler SSTs (of ~ 1 °C^49,50^), would deliver moisture that is ~ 0.5 ‰ more depleted. Other studies have emphasised other possible causes of the depleted δ^18^O values in the region, including changing rainfall seasonality^51^ and precipitation amount^51,52^. Future studies comparing the oxygen isotope synthesis presented here with isotope-enabled climate model simulations could elucidate the drivers of δ^18^O excursions during the 8.2 ka event.

Other events during the Holocene epoch are clearly less globally prominent than the 8.2 ka event. A significant number of abrupt events were identified at ~ 11.2 ka, associated with the Preboreal Oscillation, supporting the idea that freshwater pulses induced large changes in the climate system. However, the global fingerprint of this event is less coherent than that of the 8.2 ka event. Although there appear to be some statistically significant speleothem isotope excursions in the second part of the Holocene, none of these correspond to abrupt climate events that have been identified in other studies, such as the 4.2 ka event^4,5^. There is still little consensus about the spatial extent and cause of these later Holocene events, which have been interpreted as a response to changes in solar irradiance^53,54^ or volcanicity (or both)^32,55^ and which have also been considered as a manifestation of internal (unforced) climate variability^56^. The lack of a significant 4.2 ka event in our speleothem analysis could reflect the complexity of the event; records of the event do not always show a well-constrained timing^4,57,58^ or a signal with an amplitude larger than the noise of the record^59^ Furthermore, the signal of this event sometimes consists of several oscillations rather than one straightforward excursion^4,60^. However, it seems more likely that this event was not of global extent. There is no regional 4.2 ka event in the north Atlantic region^61^ and even in the Mediterranean region, where evidence of the event is clearest^4^, there are numerous palaeoclimate records that do not show the event^57^.

## Conclusion

We have shown that the 8.2 ka event is the most prominent abrupt climate event in the Holocene. The event shows a globally extensive, coherent and synchronous climate response. The coherency of the regional δ^18^O anomalies indicates that the freshwater pulse at 8.2 ka triggered a widespread reorganisation of climate systems. The synchronicity of isotope signals globally suggests that the North Atlantic freshening was transmitted via rapid atmospheric teleconnections. We have provided the first global speleothem isotope synthesis of the 8.2 ka event, that can be used to test the ability of climate models to simulate the impacts of ice sheet melting and ocean circulation changes.

## Methods

### Holocene abrupt event detection analysis

We determine the presence and timing of abrupt events during the Holocene using a global dataset of speleothem δ^18^O records from the SISAL (Speleothem Isotopes Synthesis and Analysis) version 2 database^25,26,62^. We identify abrupt events during a moving 1000-year window (with 50% overlap). For each window, we select speleothem records using the following criteria:

- They have a mean sampling resolution of <  = 30 years within the window;
- They have a minimum length within the window of 500 years;

This resulted in the selection of 275 speleothem records from 170 sites for this analysis (Fig. 1). The choice of a minimum resolution of <  = 30 years gives a minimum of five data points for abrupt events of ~ 150 years duration and ensures that there are sufficient records included in the analysis to identify a global signal.

To detect abrupt events within a window objectively, we carried out breakpoint analysis using the Strucchange package in R^63,64^. The method detects significant shifts in speleothem δ^18^O data using a dynamical programming approach. The optimal number of breakpoints (and location) is determined using a Bayesian Information Criterion. Where two breakpoints occur within < 300 years, it suggests an abrupt event, whereby there is a rapid shift in δ^18^O values, which are maintained for at least a few years, then a shift back. To prevent the breakpoint analysis from detecting changes in δ^18^O values that relate to long-term changes, the speleothem records are first individually detrended and normalised by fitting a linear regression through each record across the 1000-year window, then subtracting the predicted δ^18^O values from the linear model from the observed δ^18^O values.

We calculate the number of records within 300-year bins across the Holocene that show >  = 2 breakpoints (Fig. 2), given as a percentage of the total records in that bin (thereby ensuring plotted values do not reflect the changing number of records through the Holocene). 300-year bins are chosen to examine the presence of abrupt events because they are sufficiently short to exclude multi-centennial scale variability but long enough to capture the full length of an abrupt event. As a further step, we determined which bins were statistically significant above randomly generated noise. This was important because speleothem δ^18^O records often have a high degree of autocorrelation, which can cause statistically spurious breakpoints to be detected^65^. We therefore carried out the same breakpoint analysis on randomly generated records with the same sampling resolution and autocorrelation as the speleothem records, using the arima.sim function in R. We carried out these steps 1000 times and calculated the mean percentage of randomly generated records with >  = 2 breakpoints within a bin. Bins where the actual speleothem records have a higher percentage than the randomly generated noise are considered statistically significant.

### 8.2 ka anomalies

To identify the presence of the 8.2 ka event, and characterise the timing, duration and magnitude of speleothem oxygen isotope anomalies at the event, we selected speleothem records from SISAL version 2 covering the interval 7.8 to 8.4 ka. Records were selected using the following criteria:

- They have a mean temporal resolution of <  = 30 years within the period;
- They are at least 300 years long within the period;

This resulted in the selection of 67 speleothem records from 48 sites for this analysis (Fig. 1). We added a further 6 records (from 6 sites), that are not available in SISALv2 (Table S1). Although we used a mean temporal resolution of <  = 30 years to select the records for this analysis, 95% of records have a mean resolution of < 20 years and 67% have a mean resolution of < 10 years (Table S1).

**Figure 5:**
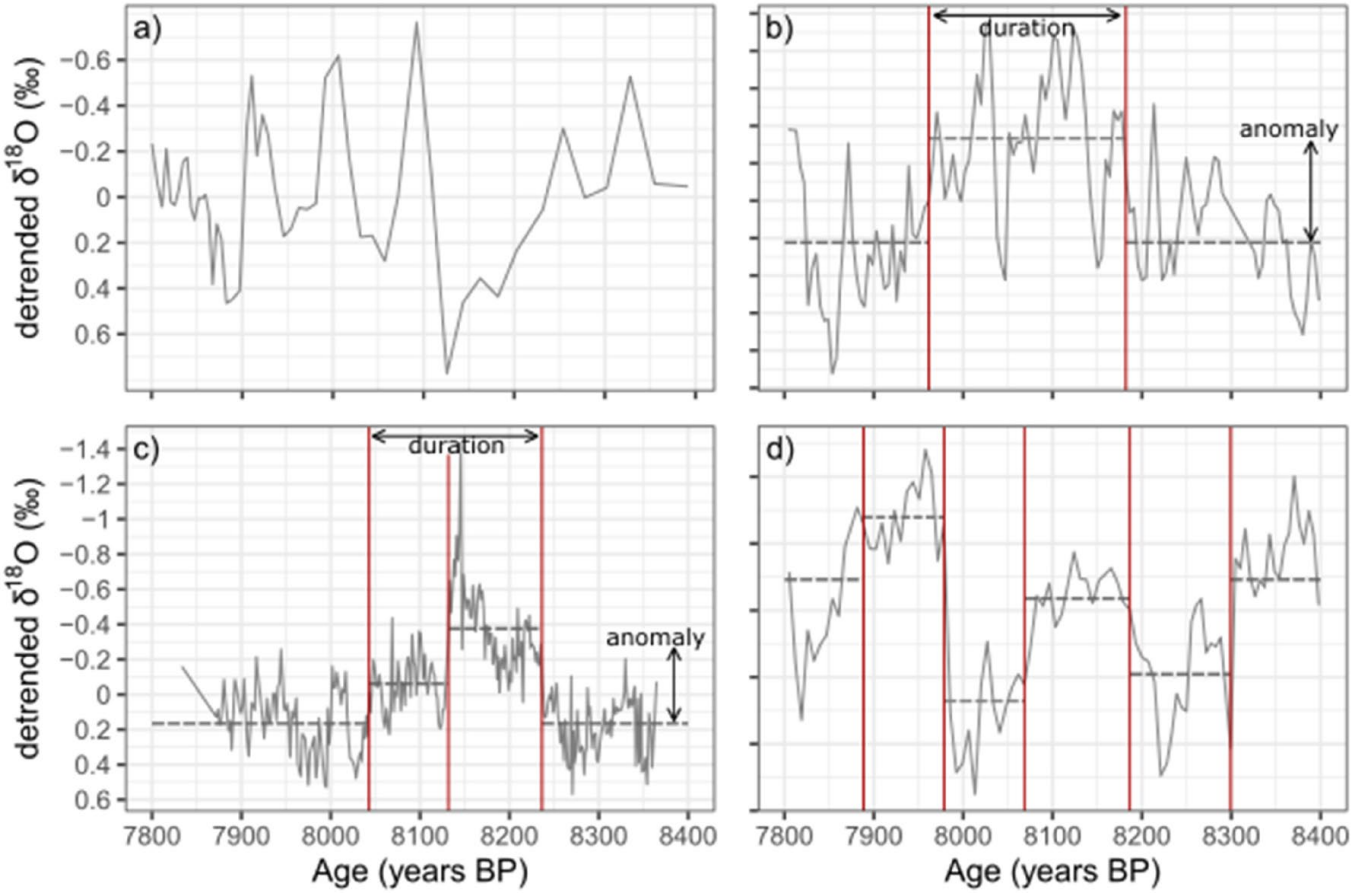
Detection of 8.2 ka isotope excursions. (**a**) shows a record (KOT-1 from Kotumsar cave^68^) with no breakpoints and therefore no excursion, (**b**) shows a record (K1 from Katerloch cave^69^) with an excursion constrained by 2 breakpoints, (**c**) shows a record (YD01 from Pippikin Pot^70^) with an excursion constrained by > 2 breakpoints, (**d**) shows a record (So-1 from Sofular cave^71^) with > 2 breakpoints, but isotope excursions of varying direction and therefore no clear 8.2 ka event.

We detected abrupt shifts in δ^18^O values by carrying out breakpoint analysis over each individual, detrended record. Where a speleothem record shows <  = 1 breakpoint, there is no significant δ^18^O excursion (Fig. 5a). Where a speleothem record shows 2 breakpoints, the record shows one simple δ^18^O excursion (Fig. 5b). If a record shows > 2 breakpoints (i.e. an excursion can be separated into segments, or there are several significant fluctuations), we determine which segments are significantly different from the base period (before and after the event). If all significant segments have the same sign anomaly (relative to the base period), this suggests they represent an event with a more complex evolution (Fig. 5c). If significant segments show a changing sign of anomaly, this suggests that the record shows numerous fluctuations and therefore the record does not show one clear 8.2 ka excursion (Fig. 5d).

The oldest breakpoint is defined as the start of the excursion and the youngest breakpoint defined as the end (Fig. 5b). We calculate the event duration in each individual record as the difference between the start and the end of the event. Anomalies are calculated as the mean δ^18^O between the start and the end of the event, minus the mean δ^18^O of before and after the event (Fig. 5).

To ensure no spurious results related to the record noise are included in our synthesis, we removed any records where the amplitude of the ~ 8.2 ka δ^18^O anomaly is smaller than one standard deviation of the base period (before and after the event), to minimise the impact of noise in the records which might result in spurious event detection.

We also examined the age uncertainties associated with the 8.2 ka δ^18^O excursions using age uncertainty data available in the SISAL v2 database. The database contains chronologies from seven different age-depth modelling approaches^26^. Age uncertainties were calculated from the spread of individual ensembles in six modelling approaches (linear interpolation, linear regression, Bchron, Bacon, OxCal, COPRA). Age uncertainties were obtained in the seventh model (StalAge) through a Monte Carlo approach, but individual ensembles were not preserved. SISAL v2 age-depth models were screened to ensure reliability. The models had to meet several criteria to be included in the database, including: (a) having no age reversals, (b) flexibly following clear growth rate changes and (c) showing greater uncertainties between dates and near growth hiatuses. No age-depth model is successful for all speleothem records. The ages (and uncertainties) were extracted at the breakpoint locations using all available age-depth modelling approaches for each record, thereby assessing uncertainty associated with the choice of model. This demonstrated whether a δ^18^O excursion could plausibly have occurred at ~ 8.2 ka. Where age uncertainties are too large and therefore the timing of the 8.2 ka isotope excursion too poorly constrained, we excluded the entity from our analysis.

## Supplementary Information

Supplementary Information.

## Data Availability

The Speleothem Isotopes Synthesis and AnaLysis (SISAL) database version 2 is available through the University of Reading Data Archive, at https://doi.org/10.17864/1947.256. ^62^. The dataset generated in this study is available in a GitHub repository, available at https://doi.org/10.5281/zenodo.5871176. We use R for analyses^66^. The code used to run the analyses and generate the figures in this study are also available at https://doi.org/10.5281/zenodo.5871176.
