# Supplementary material for: The timing, duration and magnitude of the 8.2 ka event in global speleothem records
Source: Sci Rep. 2022 Jun 22;12:10542. doi: 10.1038/s41598-022-14684-y (PMC9217811; doi:10.1038/s41598-022-14684-y)
Supplement: Supplementary file 1 — Supplementary Information. [file 41598_2022_14684_MOESM1_ESM.pdf]

Supplement to "The timing, duration and magnitude of the 8.2 ka event in global speleothem records"

Fig S1: Spatial distribution and timing of $\delta^{18}O$ anomalies for periods in the Holocene when a significant number of abrupt excursions are identified.

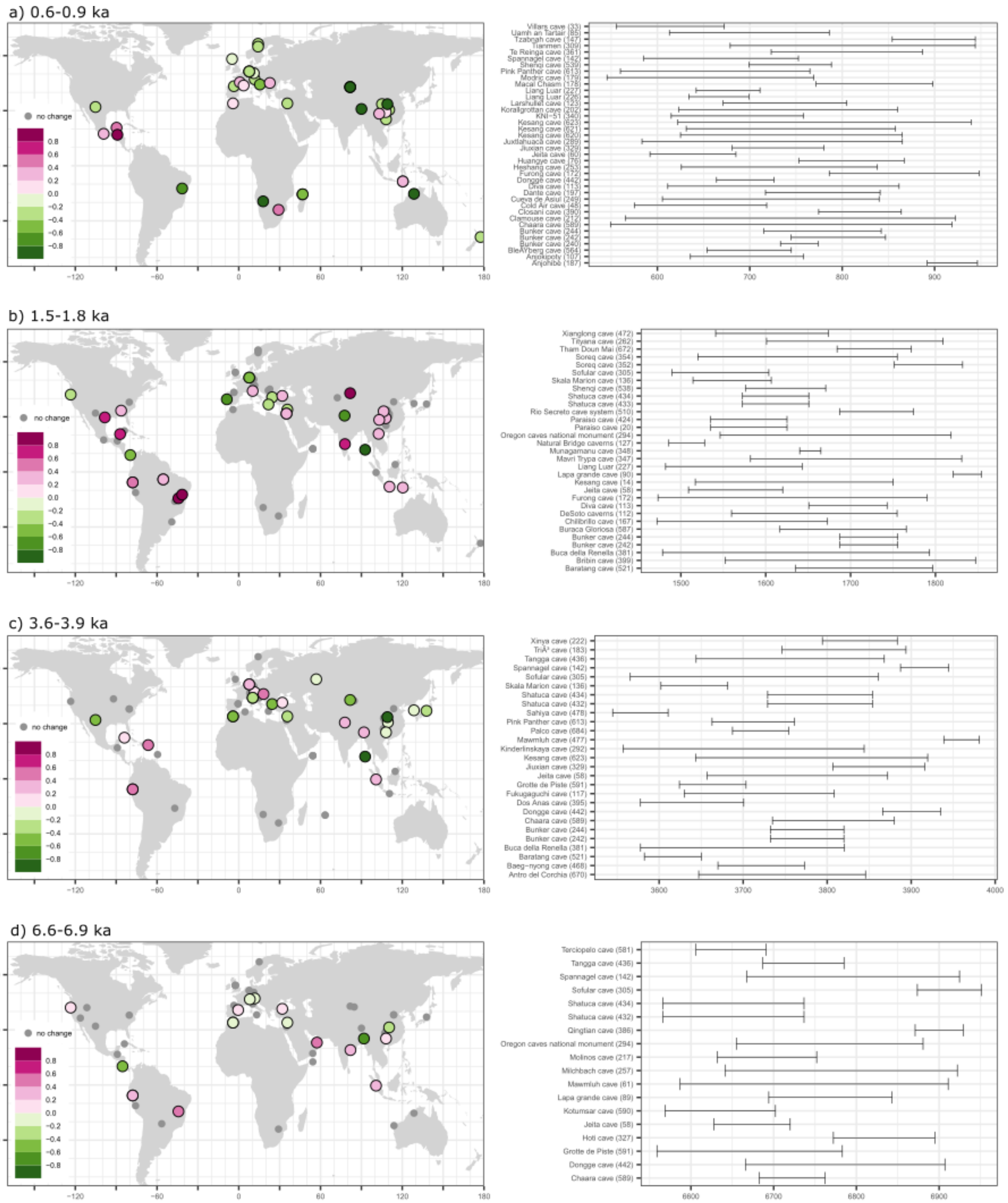

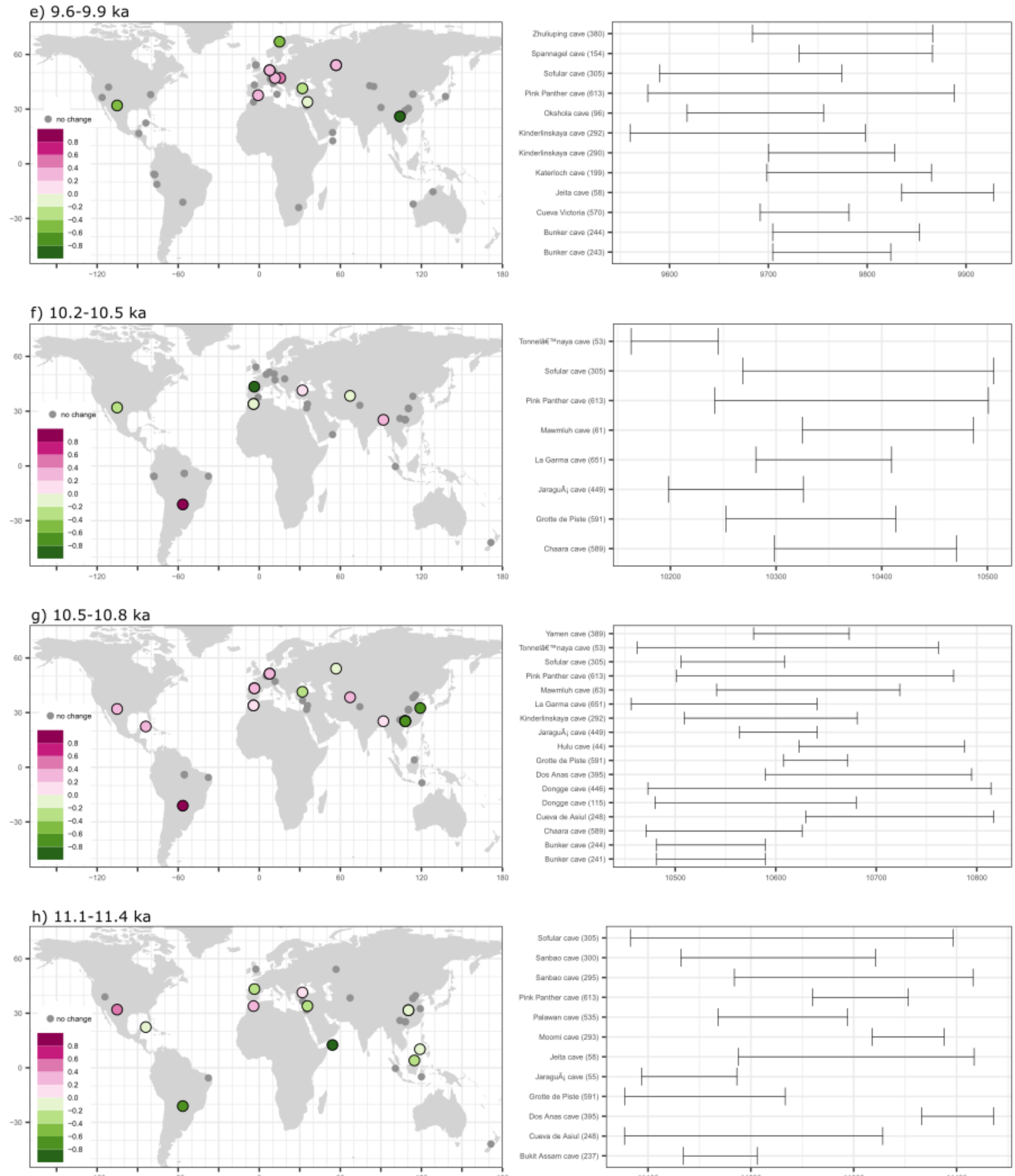

e) 9.6-9.9 ka
no change
Zhuliuping cave (380)
Spannagel cave (154)
Sofular cave (305)
Pink Panther cave (613)
Okshola cave (96)
Kinderlinskaya cave (292)
Kinderlinskaya cave (290)
Katerloch cave (199)
Jeita cave (58)
Cueva Victoria (570)
Bunker cave (244)
Bunker cave (243)
f) 10.2-10.5 ka
Tonnelâ€™naya cave (53)
Sofular cave (305)
Pink Panther cave (613)
Mawmluh cave (61)
La Garma cave (651)
JaraguÃ¡ cave (449)
Grotte de Piste (591)
Chaara cave (589)
g) 10.5-10.8 ka
Yamen cave (389)
Tonnelâ€™naya cave (53)
Sofular cave (305)
Pink Panther cave (613)
Mawmluh cave (63)
La Garma cave (651)
Kinderlinskaya cave (292)
JaraguÃ¡ cave (449)
Hulu cave (44)
Grotte de Piste (591)
Dos Anas cave (395)
Dongge cave (446)
Dongge cave (115)
Cueva de Asiul (248)
Chaara cave (589)
Bunker cave (244)
Bunker cave (241)
h) 11.1-11.4 ka
Sofular cave (305)
Sanbao cave (300)
Sanbao cave (295)
Pink Panther cave (613)
Palawan cave (535)
Moomi cave (293)
Jeita cave (58)
JaraguÃ¡ cave (55)
Grotte de Piste (591)
Dos Anas cave (395)
Cueva de Asiul (248)
Bukit Assam cave (237)

Table S1: 8.2 ka anomalies, timing and duration, obtained by breakpoint analysis. All oxygen isotope anomalies are larger than speleothem record measurement uncertainties.

entity id	site name	longitude	latitude	Start (years BP)	End (years BP)	Duration (years)	Anomaly (‰)	Notes
	Anjohibe cave	46.89	-15.54	8217	8069	148	-0.80	Not in SISALv2, data from [1]
520	Baluk cave	84.73	42.43	8285	8062	222	0.70	
242	Bunker cave	7.66	51.37	8030	7877	153	-0.38	
244	Bunker cave	7.66	51.37	8101	7869	233	-0.29	
588	Chaara cave	-4.25	33.96	8270	8065	205	-0.18	
589	Chaara cave	-4.25	33.96	8343	8232	111	-0.56	
442	Dongge cave	108.08	25.28	8325	8080	245	0.19	
475	Dongge cave	108.08	25.28	8257	8065	192	0.35	
395	Dos Anas cave	-83.97	22.38	8275	7851	424	-0.36	
117	Fukugaguchi cave	137.80	36.99	8170	8000	170	0.28	
277	Grotta di Carburangeli	13.16	38.17	8253	8105	148	-0.38	
591	Grotte de Piste	-4.25	33.95	8229	7968	261	0.25	
58	Jeita cave	35.65	33.95	8047	7881	166	-0.24	
	Kaite cave	-3.66	43.04	8211	8147	63	-0.73	Not in SISALv2, data from [2]
199	Katerloch cave	15.55	47.08	8182	7962	221	-0.56	
200	Katerloch cave	15.55	47.08	8135	8049	86	-0.46	
290	Kinderlinskaya cave	56.85	54.15	8238	8041	197	-0.37	
292	Kinderlinskaya cave	56.85	54.15	8223	8011	212	-0.35	
	Klang cave	98.73	8.33	8281	8183	98	0.29	Not in SISALv2, data from [3]
51	Lancaster Hole	-2.52	54.22	8199	8050	149	0.53	Removed from synthesis, age uncertainties too large
52	Lancaster Hole	-2.52	54.22	8272	7942	330	-0.62	
89	Lapa grande cave	-44.28	-14.37	8215	8124	91	-0.72	
496	Lianhua cave, Hunan	109.53	29.48	8147	8064	83	0.63	
529	Lianhua cave, Shanxi	113.72	38.17	8252	8088	164	0.90	
671	Limnon cave	22.14	37.96	8060	7965	95	-0.37	
256	Milchbach cave	8.08	46.62	8141	7918	223	-0.50	
466	Nuanhe cave	124.92	41.33	8328	8147	181	0.39	
	Padre cave	-44.05	-13.22	8213	8144	69	-1.34	Not in SISALv2, data from [4]
129	Pippikin Pot cave	-2.51	54.21	8236	8043	193	-0.41	
385	Qingtian cave	110.37	31.33	8183	8081	102	0.39	
351	Qunf cave	54.30	17.17	8245	7967	278	0.14	

	Rey Marcos cave	-90.30	15.40	8314	8155	159	-0.17	Not in SISALv2, data from [5]
388	Santo Tomas cave	-83.84	22.55	8202	8027	175	-0.46	
608	Santo Tomas cave	-83.84	22.55	8048	7896	152	-0.42	
433	Shatuca cave	-77.90	-5.70	8037	7900	137	-0.27	
434	Shatuca cave	-77.90	-5.70	8037	7900	137	-0.27	
540	Shigao cave	107.17	28.18	8272	8081	191	0.15	
142	Spannagel cave	11.67	47.08	8197	7924	274	-0.19	
308	Tianmen	90.07	30.92	8298	8217	81	0.79	
53	Tonnel'naya cave	67.23	38.40	8225	8117	108	-0.49	
640	Venado cave	-84.77	10.55	8144	8050	95	0.78	
150	White Scar cave	-2.44	54.17	8216	8143	73	-1.07	
	Wuya cave	105.43	33.82	8226	8065	161	0.73	Not in SISALv2, data from [6]
379	Zhuliuping cave	104.10	26.02	8260	8104	156	0.94	

Table S2: Speleothem records with evidence for an 8.2 ka oxygen isotope excursion (used in Fig. 4a) but where no data is available to carry out breakpoint analysis.

Site name	Latitude	Longitude	Isotope signal	Reference
Galeria Das Lâminas	39.51	39.51	negative	[7]
Magou cave	34.19	113.23	positive	[8]
Gasparee cave	10.39	-61.39	positive	[9]
Herbstlabyrinth	50.69	8.21	negative	[10]
Milandre cave	47.49	7.02	negative	[11]
Père Noël cave	50.13	5.16	negative	[12]

Table S3: Speleothem records where the 8.2 ka event has been identified from trace element, growth rate/lamina thickness or calcium isotope measurements (used in Fig. 4b). Interpretations of measurements are those made by the authors of the study.

Site name	Latitude	Longitude	Measurement	Anomaly	Interpretation	Reference
Ascunsă cave	45	22.6	Growth rate	positive	wetter	[13]
Botuverá cave	-27.13	-49.09	Sr/Ca	negative	wetter	[14]
Bunker cave	51.22	7.39	Mg/Ca	negative	wetter	[10]
Herbstlabyrinth	50.6878	8.2066	Mg/Ca		no change	[10]
Heshang cave	30.27	110.25	Mg/Ca	positive	drier	[15]
Heshang cave	30.27	110.25	Growth rate	negative	drier	[15]
Heshang cave	30.27	110.25	δ44/42Ca	higher	drier	[16]
Kaite cave	43.2	3.39	Growth rate		no change	[17]
Katerloch cave	47.5	15.33	Growth rate		no change	[18]

Limnon cave	37.9605	22.1403	Mg/Ca	negative	wetter	[19]
Nuanhe cave	41.2	124.55	Ba/Ca	negative	wetter	[20]
Père Noël	50.13	5.16	Sr, Ba, Mg	negative	drier	[12]
Père Noël	50.13	5.16	Growth rate	negative	drier	[12]

Table S4: Mean temporal resolution and age uncertainties for each speleothem record where an ~8.2 ka excursion was detected, across the 7.8-8.4 ka period. Mean age uncertainties are given for several age-depth modelling approaches. Only 20 % of the original chronologies included age uncertainty data. The remaining chronologies were constructed for the SISALv2 database [21].

entity id	site name	longitude	latitude	mean resolution	mean age uncertainty							
					interp	Bacon	Bchron	copRa	lin_interp	lin_reg	OxCal	StalAge
520	Baluk cave	84.73	42.43	25	-	233	-	205	192	95	-	157
242	Bunker cave	7.66	51.37	7	-	334	-	212	209	-	-	-
244	Bunker cave	7.66	51.37	4	-	-	-	-	-	-	-	-
588	Chaara cave	-4.25	33.96	16	-	-	68	26	23	6	-	25
589	Chaara cave	-4.25	33.96	10	-	59	104	77	68	-	-	44
442	Dongge cave	108.08	25.28	5	-	53	63	-	-	18	65	-
475	Dongge cave	108.08	25.28	3	-	-	35	-	31	20	27	30
395	Dos Anas cave	-83.97	22.38	10	397	-	308	-	-	-	-	128
117	Fukugaguchi cave	137.80	36.99	17	-	-	-	-	-	-	-	-
277	Grotta di Carburangeli	13.16	38.17	5	-	-	294	-	-	-	-	-
591	Grotte de Piste	-4.25	33.95	15	-	-	203	108	84	-	-	140
58	Jeita cave	35.65	33.95	6	-	34	54	34	31	-	77	37
199	Katerloch cave	15.55	47.08	5	-	-	-	-	-	40	-	77
200	Katerloch cave	15.55	47.08	2	-	113	86	122	108	52	-	95
290	Kinderlinskaya cave	56.85	54.15	11	-	-	46	72	63	-	-	-
292	Kinderlinskaya cave	56.85	54.15	9	-	-	-	-	-	-	-	-
51	Lancaster Hole	-2.52	54.22	14	130	-	246	226	209	44	-	202
52	Lancaster Hole	-2.52	54.22	18	-	-	-	-	810	37	-	-
89	Lapa grande cave	-44.28	-14.37	14	-	69	72	58	50	-	-	-
496	Lianhua cave, Hunan	109.53	29.48	10	-	22	40	21	19	-	87	24
529	Lianhua cave, Shanxi	113.72	38.17	16	-	-	112	71	56	25	-	59
671	Limnon cave	22.14	37.96	20	44	78	80	76	72	-	-	-

256	Milchbach cave	8.08	46.62	11	48	122	109	72	46	-	-	-
466	Nuanhe cave	124.92	41.33	3	-	-	-	-	-	-	-	-
129	Pippikin Pot cave	-2.51	54.21	3	30	77	70	91	-	26	-	-
385	Qingtian cave	110.37	31.33	1	-	-	-	-	-	-	-	-
351	Qunf cave	54.30	17.17	8	-	-	-	-	-	-	-	-
388	Santo Tomas cave	-83.84	22.55	13	400	-	-	386	365	332	-	327
608	Santo Tomas cave	-83.84	22.55	11	356	438	426	-	-	-	-	500
433	Shatuca cave	-77.90	-5.70	4	-	-	76	-	27	-	-	45
434	Shatuca cave	-77.90	-5.70	4	-	-	-	-	-	-	-	-
540	Shigao cave	107.17	28.18	20	-	176	111	27	23	-	-	104
142	Spannagel cave	11.67	47.08	2	-	-	-	-	-	-	-	-
308	Tianmen	90.07	30.92	6	-	119	-	-	85	-	-	45
53	Tonnel'naya cave	67.23	38.40	13	-	-	827	840	880	43	-	356
640	Venado cave	-84.77	10.55	18	-	442	283	445	396	-	-	-
150	White Scar cave	-2.44	54.17	9	34	105	76	106	101	-	-	81
379	Zhuliuping cave	104.10	26.02	11	-	368	349	503	-	70	-	-

References:

1. Duan, P. *et al.* The timing and structure of the 8.2 ka event revealed through high-resolution speleothem records from northwestern Madagascar. *Quat. Sci. Rev.* **268**, 107104 (2021).

2. Domínguez-Villar, D., Wang, X., Krklec, K., Cheng, H. & Edwards, R. L. The control of the tropical North Atlantic on Holocene millennial climate oscillations. *Geology* **45**, 303–306 (2017).

3. Chawchai, S. *et al.* Hydroclimate variability of central Indo-Pacific region during the Holocene. *Quat. Sci. Rev.* **253**, (2021).

4. Cheng, H. *et al.* Timing and structure of the 8.2 kyr BP event inferred from δ18O records of stalagmites from China, Oman, and Brazil. *Geology* **37**, 1007–1010 (2009).

5. Winter, A. *et al.* Initiation of a stable convective hydroclimatic regime in Central America circa 9000 years BP. *Nat. Commun. 2020 111* **11**, 1–8 (2020).

6. Tan, L. *et al.* Holocene Monsoon Change and Abrupt Events on the Western Chinese Loess Plateau as Revealed by Accurately Dated Stalagmites. *Geophys. Res. Lett.* **47**, e2020GL090273 (2020).

7. Benson, A. *et al.* A speleothem record from Portugal reveals phases of increased winter precipitation in western Iberia during the Holocene. *Holocene* **31**, 1339–1350 (2021).

8. Cai, Y. *et al.* Holocene variability of East Asian summer monsoon as viewed from the speleothem $\delta^{18}O$ records in central China. *Earth Planet. Sci. Lett.* **558**, (2021).

9. Boyd, M. Speleothems from warm climates: holocene records from the Caribbean and Mediterranean regions. (Stockholm University, 2015).

10. Waltgenbach, S. *et al.* Climate and structure of the 8.2 ka event reconstructed from three speleothems from Germany. *Glob. Planet. Change* **193**, 103266 (2020).

11. Affolter, S. *et al.* Central Europe temperature constrained by speleothem fluid inclusion water isotopes over the past 14,000 years. *Sci. Adv.* **5**, eaav3809 (2019).

12. Allan, M. *et al.* High-resolution reconstruction of 8.2-ka BP event documented in Père Noël cave, southern Belgium. *J. Quat. Sci.* **33**, 840–852 (2018).

13. Drăguşin, V. *et al.* Constraining Holocene hydrological changes in the Carpathian-Balkan region using speleothem δ18O and pollen-based temperature reconstructions. *Clim. Past* **10**, 1363–1380 (2014).

14. Bernal, J. P. *et al.* High-resolution Holocene South American monsoon history recorded by a speleothem from Botuverá Cave, Brazil. *Earth Planet. Sci. Lett.* **450**, 186–196 (2016).

15. Liu, Y. H. *et al.* Links between the East Asian monsoon and North Atlantic climate during the 8,200 year event. *Nat. Geosci.* **6**, 117–120 (2013).

16. Owen, R. A. *et al.* Calcium isotopes in caves as a proxy for aridity: Modern calibration and application to the 8.2 kyr event. *Earth Planet. Sci. Lett.* **443**, 129–138 (2016).

17. Dominguez-Villar, D. *et al.* Oxygen isotope precipitation anomaly in the North Atlantic region during the 8.2 ka event. *Geology* **37**, 1095–1098 (2009).

18. Boch, R., Spötl, C. & Kramers, J. High-resolution isotope records of early Holocene rapid

climate change from two coeval stalagmites of Katerloch Cave, Austria. *Quat. Sci. Rev.* **28**, 2527–2538 (2009).

19. Peckover, E. N. *et al.* Coupled stalagmite–Alluvial fan response to the 8.2 ka event and early Holocene palaeoclimate change in Greece. *Palaeogeogr. Palaeoclimatol. Palaeoecol.* **532**, 109252 (2019).

20. Wu, J. Y., Wang, Y. J., Cheng, H., Kong, X. G. & Liu, D. B. Stable isotope and trace element investigation of two contemporaneous annually-laminated stalagmites from northeastern China surrounding the 8.2 ka event. *Clim. Past* **8**, 1497–1507 (2012).

21. Comas-Bru, L. *et al.* SISALv2: a comprehensive speleothem isotope database with multiple age–depth models. *Earth Syst. Sci. Data* **12**, 2579–2606 (2020).
